# Effects of ethics communication in health care: a cluster randomised controlled trial

**DOI:** 10.1186/s12910-025-01270-w

**Published:** 2025-07-26

**Authors:** Brännström Margareta, Isaksson Ulf, Fischer Grönlund C.

**Affiliations:** Department of Nursing, Umeå University, Umeå, Sweden

**Keywords:** Clinical ethics support, CES, Cluster randomised study, Ethical climate, Ethics communication in groups, Ethics com study, Moral distress, Intervention

## Abstract

**Background:**

Studies show that healthcare professionals encounter ethically difficult situations in everyday clinical practice, and there is a need for interprofessional communication in organised forms. Ethics communication in groups (ECG), based on Habermas’s theory of communicative actions, is a form of support for interprofessional communications about ethical issues. The ‘one to five method’ is a practical tool for healthcare professionals with education in ethics to facilitate ECG in everyday clinical practice.

**Research aim:**

To evaluate the effects of organised ECG using the ‘one to five’ method for health care professionals concerning moral distress and ethical climate at wards with round-the-clock care compared with a control group.

**Research design:**

This was a prospective cluster randomised study with an open, non-blinded design.

**Methods:**

Nine wards with different medical specialisations at one university hospital were purposefully and then randomly allocated to an intervention group (IG) (*n* = 5) and a control group (CG) (*n* = 4). An ECG was performed monthly for six months in the intervention group. Prospective assessments were made at 3 and 6 months using the Measure of Moral Distress-Healthcare Professionals (MMD-HP), Moral Distress Thermometer (MDT), and the Swedish Ethical Climate Questionnaire (SwECQ).

**Result:**

Between-group analyses showed no significant differences in moral distress over time. Within-group analysis revealed that the intervention group scored lower moral distress concerning clinical causes at the patient level at the 3-month measurement point but returned to the same level as the control group at six months. The ethical climate was rated higher in the intervention group at 3 and 6 months.

**Conclusion:**

Participation in ECG likely fosters shared values and an enhanced ethical climate, though no significant differences in moral distress were observed. Moral distress may persist despite interventions, but open dialogue and professional growth can foster moral resilience. This study found a positive relationship between an ethical climate and participation in ethics communication groups (ECG) using the ‘one to five method.’ However, the small sample size limited statistical power. Future research should include larger-scale, multicentre studies and qualitative research to explore experiences with ECG.

**Trial registration:**

ClinicalTrials.gov: NCT05146102 (2021-11-05).

## Introduction

Ethics and ethically difficult situations are issues of concern for healthcare professionals in everyday clinical practice. Studies show that healthcare professionals need to reflect and communicate about ethical issues in organised forms [1, 2].

Clinical Ethics Support (CES) is a common concept for healthcare professionals worldwide, with differing organisations, structures, objectives and theoretical bases [3]. CES has been described as an ethical case intervention that is found to be complex since it concerns communication about ethical issues among stakeholders from various professional groups at professional and organisational levels [4]. Various forms of CES include clinical ethics committees (CEC) [5, 6], clinical ethics case consultations [3, 7], moral case deliberation (MCD) [8], ethics rounds [9], clinical ethics case reflections [10] and ethics communication in groups (ECG) [11]. CEC is described as an overarching concept with various purposes, organisations, functions, and practices that provide support to deal with ethical issues, such as developing guidelines, education, and fostering moral dialogue [3]. Clinical ethics case consultations are a type of healthcare service primarily aimed at assisting professionals and patients in clarifying and resolving ethical dilemmas [3, 7]. While CEC and clinical ethics case consultations sometimes serve an advisory role, their main focus is on promoting moral dialogue [3].

Moral case deliberation, described by Molewijk et al. [12], comprises various ethical communication methods, such as dialogical and pragmatic ethics dilemmas, socratic, and conversation methods. The different methods commonly concern healthcare professionals’ meetings to reflect on ethical issues, supported by a facilitator [8, 13]. The ethics round has been described by Silén et al. [9] as inter-professional communication using an imaginative ethics approach. Ethics communication in groups (ECG), based on Habermas’s theory of communicative actions, has the core purpose of supporting interprofessional communications to achieve common and broadened understandings of ethically difficult situations [11].

Various evaluation studies of CES have been performed. In focus-group interviews performed by Hem et al. [14] described the participants themselves as more analytic concerning ethical challenges after participating in ethics reflection groups. In a mixed methods study using interviews, observations and written reports [15], experiences of ethics reflection groups among community healthcare professionals were evaluated. The result showed experiences of improved quality, collegial support, and personal improvement. Weidema et al. [16] used interviews and an evaluation questionnaire to perform a responsive evaluation analysis study. Participating in MCD inspired critical attitudes to work practice, awareness of various perspectives, and improved cooperation and mutual support. Frank et al. [17] performed an intervention study in ambulance service using interviews and found that professionals gained new insights and self-awareness after participating in ethics rounds. A Delphi study was performed by McClimans et al. [18], where the majority ranked education, mediation, improved decision quality, and action as the most important objectives and outcomes.

In an observation study by Grönlund et al. [19], the participants in interprofessional ECG moved from frustrations and individual understandings of ethical difficulties to common and broadened understandings. In an interview study by Brännström et al. [20], healthcare professionals described ECG as an ‘ethical free zone’. They met not only as professionals but also as humans. In another interview study, Wälivaara et al. [21] found that ECG was a forum for in-depth communication that infused new insights and ethical awareness.

The ‘one to five method’ has been developed by Fischer-Grönlund et al. [11] as a support for healthcare professionals with education in ethics to facilitate ECG in everyday clinical practice. The method was based on results from previous observation studies and inspiration from Habermas’s theory of communicative actions [20, 22]. Habermas et al. [23 p 100–126, 303] have described communicative actions as an understanding-oriented dialogue towards common knowledge and values. Prerequisites for an understanding-oriented dialogue are that all participants are met as equals, have opportunities to speak, and may feel free to express ideals, assumptions, arguments, and counterarguments. Statements and expressions need to be sincere, comprehensible, and truthful. Communicative action is a communication process where the participants turn and twist on viewpoints, arguments, and counterarguments, and that continues until common understandings about the situation are achieved [23 p 100–126, 303].

Moral distress is a phenomenon experienced among various healthcare professionals [24, 25]. Jameton [26] defined moral distress as a condition with feelings of frustration, guilt, and anger caused by obstacles to give care that is according to one’s values. According to Hamric [27], moral distress is a condition that occurs when a person’s moral integrity is seriously compromised. Studies indicate that moral distress and the risk of turnover intentions may become reduced in a positive ethical climate [28–31]. Ethical climate has been defined as an organisation’s working climate, including reflection, shared perceptions, and understanding of values, practices, policies, and procedures [32, 33]. Olson [34] described the ethical climate within healthcare as shared perceptions, values, decisions, and mutual respect in relationships between managers, organisations, healthcare professionals, and patients.

The ‘one to five method’ as support for facilitating ECG has been sparsely evaluated. This study aimed to evaluate the effects of organised ethics communication in groups using the ‘one to five’ method for health care professionals concerning moral distress and ethical climate at hospital wards with round-the-clock care compared with a control group.

## Method

### Study design and setting

**Fig. 1:**
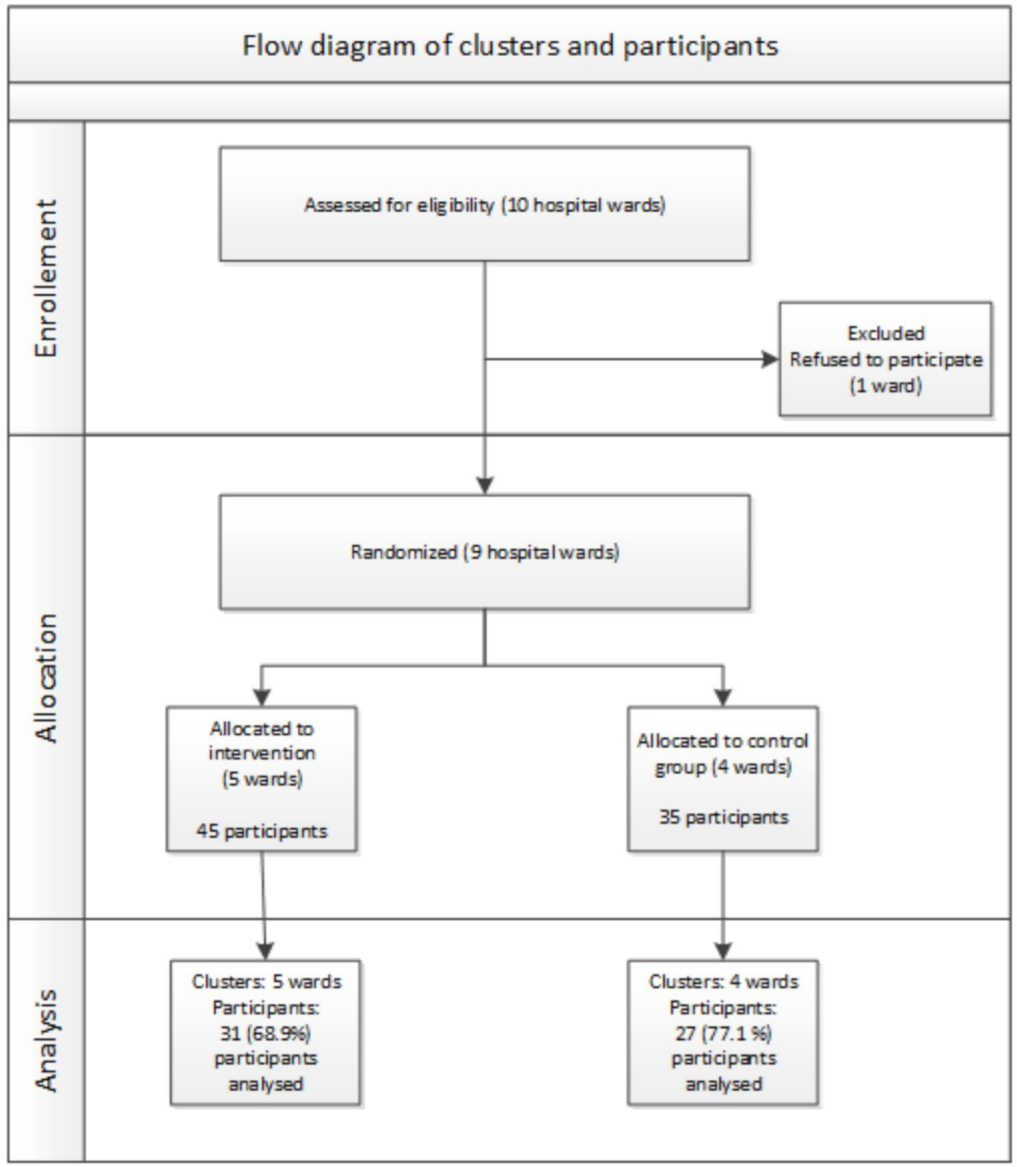
Flow diagram of clusters and participants

This study was a prospective cluster randomised study with an open, non-blinded design. The study was performed from September 2021 to May 2022. The hospital wards were chosen purposefully and then randomly allocated to an intervention and a control group (Fig. 1).

### Randomisation

Of 105 distributed questionnaires, 89 were received entirely or partially answered. Of these, 80 were completely filled out. Prospective assessments were conducted at baseline, as well as at 3 and 6 months of follow-up, with a total of 58 participants completing the survey at these three time points.

After answering the baseline questionnaires, all wards were randomly allocated to the intervention group (*n* = 5) or control group (*n* = 4) with help from an external person (not involved in the project). The specialisation for the clinics in the intervention group was psychogeriatric care, thorax intensive care, neuro-rehabilitation care specialised in neurological injuries, neuro-rehabilitation care specialised in neurological diseases, and infection care. The clinics in the control group were specialised in geriatric care, intensive care, emergency care, and palliative care.

### Inclusion criteria and participants

The inclusion criteria to participate in the study were wards with round-the-clock care and that they had employed healthcare professionals with special assignments from healthcare management concerning ethical support, namely “ethical representatives”. The “ethical representatives” should have participated in a basic ethics program arranged by the healthcare region from September 2020 to January 2021. Ten wards met the inclusion criteria and were invited, and nine wards accepted to participate in the study. Healthcare professionals from various professions– including registered nurses, enrolled nurses, physicians, physiotherapists, occupational therapists, social workers, and medical receptionists– were invited to participate by the ethical representatives or the head of the healthcare ward.

#### Intervention

The ‘one to five method’ comprises a five-step support for facilitating interprofessional ECG. The first step concerns telling the story about the situation. This means that the participants are given space to express their experience freely and put it into words. The second step consists of reflections about the emotions involved. The third step is about formulating the problem/dilemma, allowing the participants to define the value conflict and put the ethical dimension into words. In the fourth step, the analysis involves sharing knowledge and turning and twisting the perspectives to reveal a broader understanding of the situation. Finally, the fifth step concerns the choice of possible action approaches based on well-grounded arguments [11, 35].

In the intervention group (IG), healthcare professionals (*n* = 7) working as “ethical representatives” at the clinical wards went through an education program on how to facilitate ECG, supported by “the one-to-five method.” The program had a theoretical and practical approach. The ethical representatives met for a half-day education, consisting of an introduction of the theoretical base for the ‘one-to-five method’ and participating in and facilitating ECG.

After that, the ethical representatives facilitated interprofessional ECG once a month for six months at their clinical ward. The facilitators’ role was to guide the ethical dialogue supported by “the one-to-five method”. The issue of concern was an ethically difficult situation that was actual in the participant’s clinical work. The facilitator supported the participants in formulating the problem or dilemma, enabling them to identify the value conflict and articulate the ethical dimension. The participants then selected which situation would be addressed. Each session lasted for one hour and was conducted in a separate room. The number of participants in the ECG sessions varied between three and twelve. The ethical concern was meant to be further communicated and spread to the professional group. Meetings with feedback for the ethical representatives were offered once a month. However, during this period, they preferred to have only two feedback meetings because of the limited staffing situation in their wards. In the control group (CG), “ethical representatives” only participated in the basic ethics education arranged by the health care region program. They did not participate in the education program concerning ECG with the support of the “one to five method” or group meetings and did not organise ECG at their ward. Healthcare professionals from clinical wards (*n* = 4) in the control group did not participate in ECG.

### Instruments and outcomes

Prospective assessments were made at baseline, 3, and 6 months (before the first and after the third and sixth ECG) of follow-up using the Measure of Moral Distress-Healthcare Professionals (MMD-HP), Moral Distress Thermometer (MDT), and the Swedish Ethical Climate Questionnaire (SwECQ). Socio-demographic and clinical characteristics were measured at baseline using a pre-specified protocol.

The MMD-HP was developed, validated, and published by Epstein et al. [36] and translated, culturally adapted, and validated into the Swedish context [37, 38]. The instrument comprises 27 statements of ethically challenging healthcare situations or dilemmas. The items are rated according to frequency and intensity by a Likert scale from 0 (never) to 4 (very frequent) and 0 (non) to 4 (very distressing). Finally, the MMD-HP has two questions: whether a person has left or intends to leave their position because of moral distress. Each item’s frequency and intensity level (0–16) is multiplied to calculate a composite score. All items are summarised, ranging from 0 to 432– to obtain a total score. High scores indicate that the level of moral distress is high.

The Moral Distress Thermometer (MDT) is a single-tool instrument developed by Wocial and Weaver [39]. The instrument was designed as a rating scale from 0 to 10. Every second degree of the scale is associated with a descriptive word related to the degree of perceived moral stress. The descriptive wordings are rated from none, mild, uncomfortable, distressing, intense, and worst possible levels of moral distress. The respondents are asked to reflect on the perceived level of moral distress during the past two weeks. The instrument was translated, culturally adapted into Swedish, and validated [40].

The Swedish Ethical Climate Questionnaire (SwECQ), developed and validated by Grönlund et al. [41], is unidimensional and based on Habermas’ theory for a democratic dialogue. The questionnaire comprises ten items answered on a six-point scale ranging from “Not at all” (1) to “To a large extent” (6). A total score is calculated by adding the items 1–9 and the inverted item 10. A high score indicates a positive ethical climate and greater ethical communication at work. The instrument has shown good validity and reliability [41].

### Statistical analyses

With a power of 80%, a significance level of *P* < 0.05, and an estimated drop-out rate of 20%, 35 participants were needed in each arm (a total of 70 participants). Due to expected participants dropping out, the number of participants was increased to 42 in each arm (total of 84 participants).

The participants’ characteristics are presented through descriptive statistics (Table 1). The intervention and the control group were compared using Repeated Measures ANOVA. Sphericity was analysed using Mauchly’s W. If sphericity was present, it was compensated by the Huynh-Feldt or Greenhouse-Geisser test when appropriate. A *p*-value < 0.05 was considered statistically significant. Jamovi, version 2.3.18, was used in all analyses.

**Table 1:** Description of participants included in the analysis

	Total	Intervention	Control	*p*-value
Sex (*n*, %)				0.330
Female	50 (86)	28 (90)	22 (81)	
Male	8 (14)	3 (10)	5 (19)	
Age in year (*n*, %)				0.493
21–30	10 (17)	4 (13)	6 (22)	
31–40	9 (16)	7 (23)	2 (7)	
41–50	14 (24)	8 (26)	6 (22)	
51–60	18 (31)	8 (26)	10 (37)	
61 or over	7 (12)	4 (13)	3 (11)	
Occupation (*n*, %)				0.170
Assistant nurse	15 (26)	11 (37)	4 (15)	
Nurse	25 (44)	11 (37)	14 (32)	
Physician	7 (12)	2 (7)	5 (19)	
Other	11 (17)	6 (20)	4 (15)	
Years of professional experience in healthcare	18.60 (12.44)	18.00 (13.86)	19.26 (10.87)	0.575
Years of experience at current workplace (mean, SD)	9.91 (9.26)	10.19 (9.87)	9.59 (8.67)	0.808

## Results

**Fig. 2:**
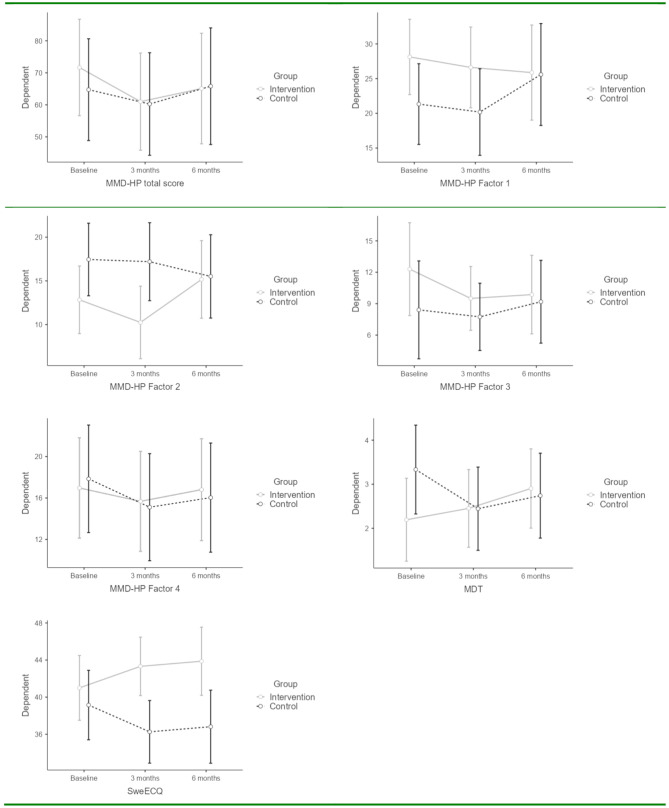
Description of the most significant effects of the intervention

The result showed no significant differences over time within the groups in either of the variables. However, a difference between the intervention and the control group could be seen concerning factor 2, related to clinical causes at the patient level, and SweECQ– the ethical climate. Concerning factor 2, the intervention group scored lower at the 3-month measurement point but returned to the same level as the control group at six months. Regarding the ethical climate, the intervention group rated the climate as higher at 3 and 6 months, although the 6-month point was not entirely significant. However, there was a trend that the intervention group rated their ethical climate increasingly better during the intervention. No other significant differences could be found (Table 2; Fig. 2).

**Table 2:** Description of the intervention’s effect. Intervention, *n* = 31, control, *n* = 27

	Baseline Mean (SD)	3 months Mean (SD)	6 months Mean (SD)	*p*-value Within groups	*p*-value Between groups	*p*-value baseline-6 months	d
MMD-HP total score (0-432)				0.315	0.699		
Intervention	71.67 (43.69)	61.00 (40.17)	66.94 (49.83)			0.516	− 0.14
Control	64.74 (38.19)	60.26 (42.94)	65.81 (44.36)			0.857	0.03
MMD-HP System level (0-128)				0.491	0.203		
Intervention	28.13 (16.31)	26.61 (16.39)	25.87 (18.93)			0.549	− 0.13
Control	21.33 (13.52)	20.19 (15.92)	25.59 (19.16)			0.156	0.25
MMD-HP Patient level (Clinical causes) (0–96)				0.253	0.040		
Intervention	13.10 (9.40)	10.23 (9.91)	15.35 (13.57)			0.295*	0.19
Control	17.15 (11.44)	17.22 (13.15)	15.30 (10.27)			0.328	− 0.17
MMD-HP Perceived integrity related to colleagues (0-112)				0.513	0.546		
Intervention	12.30 (15.23)	9.50 (8.89)	10.29 (11.31)			0.618*	− 0.18
Control	8.41 (7.20)	7.74 (7.65)	9.19 (9.02)			0.600	0.09
MMD-HP Interactions with patients and families (0–96)				0.381	0.823		
Intervention	16.97 (12.32)	15.68 (12.96)	16.81 (14.16)			0.948	− 0.01
Control	17.85 (14.32)	15.11 (13.92)	16.04 (13.09)			0.218	− 0.13
Moral Distress Thermometer (0–10)				0.552	0.537		
Intervention	2.19 (2.33)	2.45 (2.35)	2.90 (2.57)			0.289	0.29
Control	3.33 (2.91)	2.44 (2.56)	2.74 (2.41)			0.295*	− 0.22
Ethical climate (0–60)				0.859	0.036		
Intervention	41.00 (11.64)	43.32 (9.58)	43.87 (9.15)			0.120*	0.27
Control	39.15 (6.84)	36.26 (7.68)	36.81 (11.32)			0.184	− 0.25

* = Wilcoxon

## Discussion

The main findings of this study showed a trend of improved ethical climate in the intervention group during the intervention at 3 and 6 months. However, there were no significant differences over time concerning the level of moral distress. The baseline moral distress was reasonably low, which may explain the lack of change in the outcome of moral distress.

The result showed that the ECG and sharing perspectives among professionals positively impacted the ethical climate. Similarly, Okumoto et al. [42] found significant improvements in the ethical climate following interventions in ethics education and training. In contrast, Silén et al. [43] found no impact on the ethical climate from ethics rounds interventions. According to Lützen et al. [44], both the ethical climate and the individual´s moral sensitivity may influence the intensity of moral distress.

Notably, the intervention in this study had no impact on reducing the experience of moral distress after six months. Previous intervention studies have shown varying effects on moral distress. For example, interventions such as empowerment programs, education, and open dialogue led to decreased moral distress [45–47]. However, other interventions, such as moral distress consultation services (MDC), had a marginal effect on moral distress [48]. In a study by Leggett et al. [49], the participants scored a temporary increase in moral distress immediately after an educational intervention.

Our findings support previous research showing that interventions promoting ethical reflection and dialogue, such as ECG, can enhance perceptions of the ethical climate, even if the impact on moral distress is limited. This is consistent with Ashida et al. [50], who found that moral competence improved through ethical support programs, despite no significant reduction in moral distress. Epstein et al. [48] emphasize that ethics consultations may not directly alleviate moral distress but can help healthcare professionals identify ways to address morally challenging situations. Lützén and Ewalds-Kvist [51] reflected on the relationship between moral distress, moral sensitivity, and moral resilience, noting that moral sensitivity can trigger moral distress when a person cannot act according to one’s moral agency. It was argued that interprofessional dialogue among involved professionals may help make meaning of experiences and foster an atmosphere conducive to moral resilience. Our study aligns with this perspective, as moral distress persisted over time, yet participants in the intervention group reported a more positive ethical climate, something that may contribute to moral resilience.

Furthermore, our findings reflect the conclusions of Coremans et al. [52], who observed that experiencing moral distress in a positive ethical climate with open dialogue fostered shared perspectives, learning opportunities, professional growth, and achieved moral resilience. Similarly, our study suggests that participation in ECG can create space for collective reflection and support the development of strategies for handling ethical challenges, even in the absence of a sustained reduction in moral distress. Young and Rushton [53] have also challenged the predominantly negative framing of moral distress, proposing a more nuanced concept that includes the potential for growth and resilience. This perspective is echoed in our interpretation of the results: that open dialogue and collegial reflection within ECG may help healthcare professionals develop the capacity to face morally distressing situations.

This study was performed during the COVID-19 pandemic, a time marked by large-scale, ethically difficult healthcare situations. During this period, professionals in the intervention group rated slightly decreased moral distress after three months, while professionals in the control group rated increased moral distress. This raises the question of whether the ECG was a protective factor against moral distress.

Previous intervention studies showed ECG as a forum for healthcare professionals to address ethically difficult situations from various perspectives and reach a shared understanding [19, 21]. The ‘one to five method’ is inspired by Habermas’ theory of communicative actions. He [54] linked the lifeworld to our intuitive pre-understandings, values, norms, attitudes, and resources as crucial when interpreting situations. Communicative actions reveal various intersubjective experiences and reference systems leading to shared interpretations and knowledge [54 p 133–139]. According to Benhabib [55], communicative ethics presupposes a value differentiation process that opens up for argumentation among humans with various lifestyles, beliefs, and values. Moral reflexivity is to take a step beyond one’s conventional morality and open up for a common ideal of humanity [55 p 62–65]. This study showed no change regarding moral distress during the intervention, either between groups or over time. However, the results indicated a positive relationship between ECG participation and improved ethical climate, which may reflect the natural development of moral awareness through communication that fosters shared interpretations, beliefs, and values.

### Methodological considerations

The sample size in this study was small, and full statistical power was not achieved. As a result, the findings should be generalised with caution. The intervention and data collection was performed during the COVID-19 pandemic, during which healthcare professionals faced new challenges and ethically difficult situations that might have influenced their experience of moral distress. Some of the wards included in the study were repurposed to care for patients suffering from COVID-19, while others specialised in caring for patients with serious illnesses who were prioritised for care but typically did not belong to those specific wards. Dunham et al. [56] noted that healthcare professionals encountered new ethical challenges during the pandemic. On the one hand, they had to navigate tensions between clinical and public health ethics; on the other, they had to provide care beyond their expertise with limited resources. Spilg et al. [57] found that healthcare professionals working with patients suffering from covid 19 experienced increased moral distress. These demanding conditions may have contributed to the low and decreasing response rate in this study and to the unchanged levels of moral distress after 3 and 6 months. While the intervention in this study was found to have a positive effect on the ethical climate, this outcome should also be interpreted with caution, considering the small sample size.

During the pandemic, external visitors were not permitted to visit the wards to prevent the spread of infection. This posed a challenge for the researchers in reaching out to healthcare professionals and obtaining information or reminders from them. This could be another reason for the study’s low response rate. The completeness of the returned questionnaires varied. Most respondents completed the MDT and SwECQ, but several MMD-HP questionnaires were only partially filled in and excluded from the analysis. The MMD-HP is a comprehensive questionnaire requiring considerable time and focus to complete. The stressful conditions in clinical settings during the COVID-19 pandemic may have contributed to the incomplete responses.

In this study, the facilitators in the intervention group consisted of healthcare professionals working as “ethical representatives”. This can be considered a strength, as it ensured that the researchers did not directly influence the participants. The intervention was performed over a limited period, which may have been too short to observe any significant changes in participants’ experiences of moral distress. Further, more extensive multicentre studies are needed to explore how interprofessional ethics communication in groups may impact moral competence among healthcare professionals.

## Conclusion

This study found a positive relationship between the experience of an ethical climate and participation in ethical communication in groups (ECG), facilitated by ethical representatives on the ward using the “one to five method.” Participation in ECG likely fosters shared understandings and values, which, in turn, contribute to an enhanced ethical climate. However, no significant differences were observed between the groups, nor was there any change over time regarding the experience of moral distress. Participation in ECG may raise moral awareness in situations involving conflicting values, which could explain why moral distress persists. Given the relatively small sample size in this study, full statistical power was not achieved. Future research should include larger, multicenter studies to better understand the ECG’s impact on moral distress. Furthermore, the effects of moral competence and resilience among healthcare professionals need to be investigated. Additionally, qualitative research is needed to explore the experiences of facilitators and participants with ECG, especially in the context of the “one to five method.”

## Data Availability

The datasets used and analysed during the current study are available from the corresponding author upon reasonable request.

## Abbreviations

CES: Clinical Ethics Support
CG: Control group
ECG: Ethics communication in groups
IG: Intervention group
MCD: Moral case deliberation
CEC: Clinical ethics committes
MMD-HP: Measure of Moral Distress-Healthcare Professionals
MCD: Moral distress consultation services
MDT: Moral Distress Thermometer
SwECO: Swedish Ethical Climate Questionnaire
